# Pitfalls in the genetic testing of the *OPN1LW-OPN1MW* gene cluster in human subjects

**DOI:** 10.1038/s41525-024-00406-y

**Published:** 2024-05-04

**Authors:** Bernd Wissinger, Britta Baumann, Elena Buena-Atienza, Caspar Gross, Susanne Kohl

**Affiliations:** 1 Molecular Genetics Laboratory, Institute for Ophthalmic Research, Centre for Ophthalmology, University of Tübingen, Tübingen, Germany; 2 Institute for Medical Genetics and Applied Genomics, University of Tübingen, Tübingen, Germany; 3 NGS Competence Center Tübingen, Tübingen, Germany

**Keywords:** Molecular medicine, Retinal diseases

**arising from** Haer-Wigman, L. et al. *npj Genomic Medicine* 10.1038/s41525-022-00334-9 (2022)

We read with much interest the recent publication in this journal by Haer-Wigman and colleagues on the diagnostic analysis of the *OPN1LW-OPN1MW* gene cluster^1^. We appreciate the adaptation and implementation of novel technologies like Multiplex Ligation-Dependent Probe Amplification (MLPA), Long-Read Sequencing, and Optical Genome Mapping (OGM) applied by the authors to address the difficulties in genetic diagnostic analysis of the *OPN1LW-OPN1MW* gene cluster. These difficulties are due to the high sequence similarity of the *OPN1LW* and *OPN1MW* genes including intronic as well as intergenic sequences, shared sequence variants occurring in both genes, the presence of *OPN1LWxOPN1MW* or *OPN1MWxOPN1LW* hybrid genes, and gene copy number variability. In fact, the *OPN1LW-OPN1MW* gene cluster is a series of segmental duplications (SDs) arranged as tandem low copy repeats with a unit size of about 39 kb^2^. Prior studies have shown that the probability of expression of a specific gene copy within the *OPN1LW-OPN1MW* gene cluster depends on its distance to the upstream locus control region (LCR), an enhancer element which is essential for the expression of the *OPN1LW* and *OPN1MW* genes^3,4^. This gradient in expression of gene copies is responsible for that only the first two, most proximal gene copies are relevant for the color vision or cone dysfunction phenotype linked to deleterious variants or structural rearrangements in the *OPN1LW* and/or *OPN1MW* genes^5^. Thus, the actual order of gene copies and the corresponding location of deleterious variants is of eminent importance for a reliable and non-ambiguous genetic diagnostic in subjects with more than two opsin gene copies. The latter is rather common. In fact, we observed a mean opsin gene copy number of 3.31 for unaffected males^6^, and we encounter more than two gene copies in the majority of subjects for which genetic testing of the *OPN1LW-OPN1MW* gene locus is inquired in our laboratory based on a provisionally clinical diagnosis of cone dystrophy, achromatopsia, Bornholm Eye Disease (BED) or Blue Cone Monochromacy (BCM). We therefore well appreciate any technical progress that enables to decipher the exact order of distal *OPN1MW* or *OPN1MW/LW* hybrid gene copies.

Haer-Wigman and colleagues proposed a strategy of long-read sequencing of a set of long range PCR amplicons which cover i) the first most proximal gene copy, ii) non-discriminating, the second and all consecutive gene copies, and iii) the last, most distal gene copy. For the latter they used PCR primers of which the reverse primer is said to be unique *“…Because the reverse primer of this amplicon is located outside the duplicated region and because in all samples only one of the two earlier detected (hybrid) OPN1MW genes was sequenced* (Supplementary Table 1)*, we assume that the amplicon is specific for the last opsin gene of the cluster. …”*. We disagree with this statement since the binding site sequence of their reverse primer is well located within the segmental duplication (SD) region of the *OPN1LW-OPN1MW* gene cluster although within a 697 bp insertion (Fig. 1, Supplementary Fig. 1, and denoted as SDIns in the following) which has been mapped to the last-but-one SD copy of the gene cluster in the hg38 genome assembly and the terminal SD copy of the gene cluster in the T2T CHM13.2.0 assembly.

**Fig. 1:**
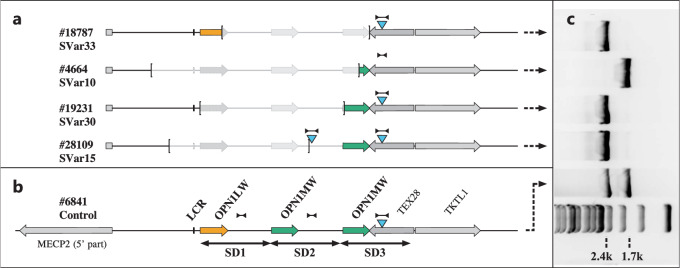
Presence of the SDIns in BCM patients with partial deletions of the *OPN1LW-OPN1MW* gene cluster. Schematic representation of the *OPN1LW-OPN1MW* gene cluster in patients with partial deletions with the extent of the deletions marked by brackets (**a**) in comparison with a normal control (**b**) with one *OPN1LW* gene (red arrow) and two *OPN1MW* gene copies (green arrows). SD1, SD2 and SD3 indicate the extent of individual segmental duplications (SD) forming the opsin gene cluster. The blue triangles indicate the presence and localization of the 697 bp SDIns. The long distance PCR amplicon LD-SDIns covering the region of SDIns and flanking sequences which was used to distinguish between presence (2.4 kb PCR product) or absence (1.7 kb PCR product) of the SDIns is indicated by dumbbell-shaped marks. The agarose gel separation of the long distance PCR products for the patients with deletions and the control is montaged alongside (**c**) including a 1 kb DNA ladder size standard at the bottom. Note that the presence of two copies of the SDIns in proband #28109 was further supported by qPCR.

We therefore wondered whether this SDIns – and thus the primer binding site crucial for the long range PCR amplicon in the Haer-Wigman *et al*. paper – is truly specific for the terminal gene copy or rather an insertion polymorphism in the SD sequence. We first tested for the presence and the copy number of the SDIns in four BCM patients with precisely mapped deletions which encompass upstream parts of the *OPN1LW-OPN1MW* gene cluster up to the most distal SDs or parts thereof^7^. Notably, we observed one proband (#4664) lacking the SDIns in the terminal SD copy and one proband (#28109) in which both the terminal copy and the non-deleted part of the last-but-one SD copy carry the SDIns (Fig. 1).

The deletion in proband #4664 is a de novo mutation in the maternal grandfather’s germline as reported previously^8^. In order to rule out a loss of the SDIns due to a more complex rearrangement in this de novo event, we investigated the *OPN1LW-OPN1MW* gene cluster in the maternal grandfather (#5762) from whom the chromosomal segment was inherited (Fig. 2). Proband #5762 has an *OPN1LW-OPN1MW* gene cluster with a total of two gene copies, a single *OPN1LW* gene followed by a single *OPN1MW* gene copy. We confirmed the absence of the SDIns in the distal *OPN1MW* gene SD copy (as observed in the grandson), but its presence in the proximal *OPN1LW* gene SD (Fig. 2).

We then went on to screen for the presence and the copy number of the SDIns in a larger series of color vision normal subjects (*n* = 15) and clinical cases (*n* = 37, including 14 male probands with protan or deutan color vision defects and 23 male probands with a relevant clinical diagnosis of BCM, BED, cone dystrophy or achromatopsia) with three or more gene copies in the *OPN1LW-OPN1MW* gene cluster, where the order of the distal gene copies is difficult to determine by current technology. We found that 53.8% (28/52) of the probands in this cohort do not obey the proposed rule, i.e. presence of the SDIns exclusively in the terminal SD copy (Table 1). The observed tendency of two or more copies of the SDIns in clinical cases (60.8% versus 26.6% in non-affected probands) is noteworthy but needs further exploration in a larger cohort.

**Fig. 2:**
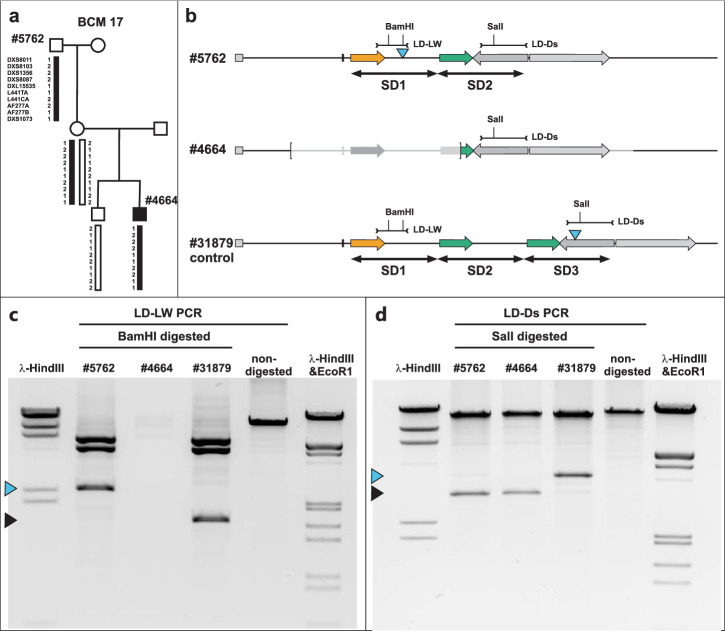
Sole presence of the SDIns in the *OPN1LW* gene in family BCM 17. **a** Pedigree of family BCM 17 with the grandson (#4664) affected by BCM due to a de novo deletion at the *OPN1LW-OPN1MW* gene locus in the grandfather’s (#5762) germline. Inheritance of the X chromosome segment covering the *OPN1LW-OPN1MW* gene luster was confirmed by microsatellite marker analysis (adapted from Buena Atienza et al. 2018). **b** Structure of the *OPN1LW-OPN1MW* gene array in #5762 (grandfather) and #4664 (affected grandson) with the extent of the deletion in the latter indicated by brackets in comparison with a control subject (#31879). SD1, SD2 and SD3 indicate the extent of individual segmental duplications forming the opsin gene cluster. The blue triangles indicate the presence and localization of the 697 bp SDIns. The long distance PCR amplicons covering the region of SDIns in SD1 (LD-LW) and the most distal SD (LD-Ds) are indicated by dumbbell-shaped marks. Cleavage sites for restriction enzymes *Bam*HI and *Sal*I in these amplicons are indicated. **c**, **d** Agarose gel separation of *Sal*I-digested LD PCR amplicon LD-LW and *Bam*HI-digested LD-PCR amplicon LD-Ds, respectively, for subjects #5762, #4664, and #31879. Blue and black triangles mark the RFLP fragments covering the SDIndel site, differentiating between the presence (blue triangle) or absence (black triangle) of the 697 bp insertion. *Hin*dIII or *Hin*dIII and *Eco*RI digested phage λ DNA was used as size standards.

**Table 1:** Copy number of the SDIns polymorphisms in our study cohorts

	No. of probands with 0, 1 or ≥2 copies of SDIns		
	0 copy	1 copy	≥2 copies
Centromeric deletions (*n* = 4)	1	2	1
Color vision normal (*n* = 15)	2	9	4
Color vision or cone deficient (*n* = 37)	2	15	20
Σ	5	26	25

Fully compliant with the result of our screening approach, we verified by Oxford Nanopore Cas9-based targeted long-read sequencing the presence of two copies of the SDIns – one located in the most proximal gene copy and the other located in the most distal gene copy – in a color vision deficient subject carrying a total of four gene copies (Fig. 3).

**Fig. 3:**
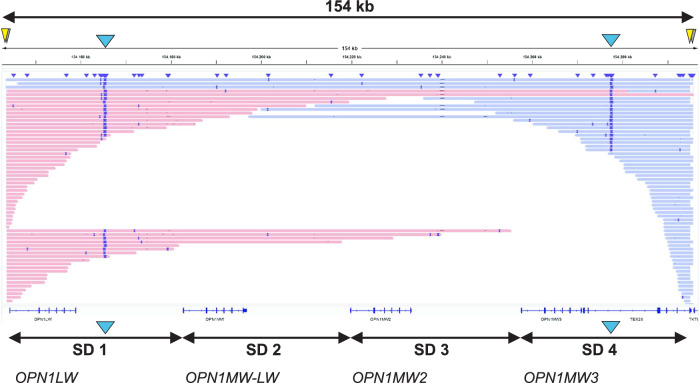
Cas9-based targeted long-read sequencing of Cas9-enriched *OPN1LW-OPN1MW* gene cluster in a deuteranomalous proband with four opsin gene copies. Long reads mapped to hg38 are visualized in IGV and colored by read strand. A total of four CRISPR/Cas9 excision cuts (yellow and shaded triangles) were designed to specifically capture the complete *OPN1LW-OPN1MW* gene cluster. The sequences at the excision sites were used to guide a custom alignment to the hg38 reference. A total of two copies of the SDIns (blue triangle) were found in this subject, one in the most proximal SD copy (including an *OPN1LW* gene) and another in the most distal SD copy (including an *OPN1MW* gene). The SDIns copies in SD1 and SD4 are covered by 24 and 21 long reads, respectively. A total of five ultra-long reads cover the complete 154 kb sized *OPN1LW-OPN1MW* gene cluster also spanning both SDIns insertions in SD1 and SD4.

To demonstrate the misleading or inconsistent results which in some cases may arise from the strategy proposed by Haer-Wigman and colleagues, we designed long distance PCR amplicons (see Supplementary Methods and Supplementary Table 1 for primer sequences) to amplify and sequence exon 5 from the most proximal and the most distal gene copy in comparison to the sequence obtained with the said terminal gene copy amplicon (LD-X) from this prior study. In proband #5762 which harbors the SDIns in the proximal gene copy (see also Fig. 2), we observed *OPN1LW* and *OPN1MW* exon 5 sequences for the proximal and the distal gene copy, respectively, but an *OPN1LW* exon 5 sequence for the said LD-X amplicon (Fig. 4a). As a second example, we investigated proband #3327, a male patient with a clinical diagnosis of incomplete achromatopsia who was found to carry three opsin gene copies and three copies of the SDIns. Comparative exon 5 sequencing revealed *OPN1LW* and *OPN1MW* sequences for the most proximal and the most distal gene copy, respectively, but an overlay of *OPN1LW* and *OPN1MW* sequences for the said LD-X amplicon (Fig. 4b). Similarly, overlaid sequences were obtained from the said LD-X amplicon for exon 3 (Supplementary Fig. 2) which thereby precludes the determination of variant haplotypes, some of which are known to cause splicing defects^9–12^, an issue of major importance in current X-linked cone opsin gene genetic diagnostics.

**Fig. 4:**
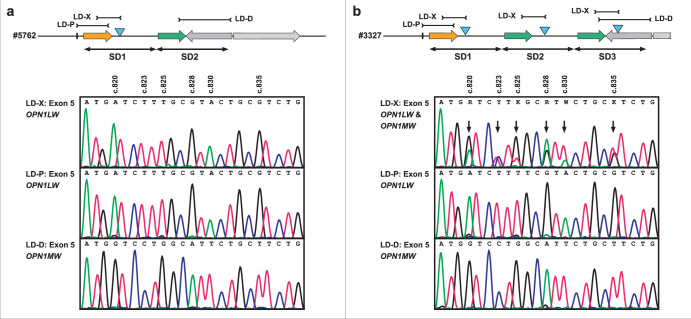
Different exon 5 sequences based on the design of LD PCR amplicons. Exon 5 sequences for proband #5762 from family BCM 17 (**a**) and proband #3327 (**b**) obtained for the LD PCR amplicon LD-X (as proposed by Haer-Wigman and co-workers) in comparison with those obtained for LD PCR amplicons LD-P (specific for the most proximal gene copy) and LD-D (specific for the most distal gene copy). Top: Deduced structure of the *OPN1LW-OPN1MW* gene cluster in the two probands. SD1, SD2, and SD3 indicate the number and extent of individual segmental duplications forming the opsin gene cluster. The blue triangles indicate the presence and localization of the 697 bp SDIns. Bottom: Sanger sequences for parts of exon 5 as obtained from the different LD PCR amplicons. Variant sites between *OPN1LW* and *OPN1MW* are indicated above the chromatograms and arrows point to sites with overlaid sequence. Note that for #3327 the LD PCR amplicon LD-X results in a mixed, overlaid sequence due to the amplification of multiple gene copies.

In conclusion, we sound a note of caution to rely on the strategy of Haer-Wigman *et al*. for the determination of the order of *OPN1LW* and *OPN1MW* copies and variants therein without prior experimental validation that the relevant SDIns is present as a single copy and solely located in the most distal SD copy of the *OPN1LW-OPN1MW* gene cluster. If not taken into account, it may lead to inconclusive or misleading interpretation of the results. To overcome the limitations of current testing assays, we rather encourage the adaptation and implementation of phasing-like SNP-based ordering of long sequencing reads from a fully tiled set of overlapping long distance PCR fragments or long-read sequencing technology as shown herein as an unconstrained approach for the genetic diagnostics in probands with a complex *OPN1LW-OPN1MW* gene cluster.

## Reporting summary

Further information on research design is available in the Nature Portfolio Reporting Summary linked to this article.

### Supplementary information

Supplementary Information

Reporting summary

## Data Availability

The data and datasets generated during the current study are available from the corresponding author on reasonable request.
