# Supplementary material for: Pitfalls in the genetic testing of the OPN1LW-OPN1MW gene cluster in human subjects
Source: NPJ Genom Med. 2024 May 4;9:28. doi: 10.1038/s41525-024-00406-y (PMC11069579; doi:10.1038/s41525-024-00406-y)
Supplement: Supplementary file 1 — Supplementary Information [file 41525_2024_406_MOESM1_ESM.pdf]

Pitfalls in the genetic testing of the *OPN1LW-OPN1MW* gene cluster in human subjects

Supplementary Methods

Subjects, biosamples and standard DNA isolation

The study was performed in compliance with the tenets of the WMA Declaration of Helsinki. The study was approved by the Ethics Committee of the Medical Faculty, Eberhard-Karls University Tübingen and consent for research was obtained from participants. Genomic DNA was isolated from venous blood samples according to standard salt-out & precipitation or column –based procedures if not stated otherwise.

Copy Number qPCR

qPCRs were done to determine the copy number of the 697 bp SDIns polymorphisms relative to *OPN1LW* and *OPN1MW* copy number and an autosomal single copy control sequence (*SDC4* locus on chromosome 20). qPCR assay primers are listed in Supplementary Table 1. qPCRs reactions (15 µl volume, 30 ng template DNA) were done in triplicates with all four assays in parallel on the same instrument run on an ABI 7500 qPCR instrument using Quantitect SYBR Green PCR Kit (Qiagen, Hilden Germany). ΔCt values (SDC4 control – test assay) from a series of subjects with defined *OPN1LW-OPN1MW* gene copies (as determined by Southern blot detection of fragment size of pulsed-field gel electrophoretic separation of *Not*I-digested genomic DNA and Fiber-FISH (Wolf et al. 1999)) and selected subjects with partial deletions of the *OPN1LW-OPN1MW* gene cluster served as calibrators for *OPN1LW-OPN1MW* gene copy number and SDIns single copy, respectively.

Multiplex Ligation-Dependent Probe Amplification (MLPA)

For MLPA we used a pre-marketing release version of the SALSA X080-B1 Opsin probe-mix (MRC Holland, Amsterdam, The Netherlands) targeting sequences of the LCR, and individual exons of *OPN1LW* and *OPN1MW* specifically. MLPA was performed according to the manufacturer's recommendations and amplification products were separated by capillary electrophoresis on an ABI 3130 instrument. Data analysis was done using the Coffalyser.Net software (MRC Holland) which calculates ratios in comparison to reference samples co-processed with the test samples.

Long distance PCR amplification and restriction enzyme digestion

Long distance PCR amplifications were done in 25 or 50 µl volumes using the NEB LongAmp HotStart Taq 2 x Master Mix (New England Biolabs, Frankfurt/Main, Germany) with 2-3 ng/µl template DNA and 400 nM of forward and reverse primers. By default, we used the following standard LD PCR cycling protocol for amplification: 1 min 94°C, 30 x [30 sec 94°C, 14-16 min 65°C], 10 min 65°C. 5-15 µl of LD PCR products were used for restriction enzyme digestion with *Bam*HI (LD-LW) or *Sal*I (LD-Ds) in a volume of 20 µl with 20 U of enzyme for 2 h at 37°C. The digested LD PCR fragments were separated on 0.8%

agarose gels.

Sanger Sequencing

LD PCR products were diluted 1:200 to 1:1000 and subjected to nested amplification with primers for exon 3, exon 4 and exon 5 of the *OPN1LW/OPN1MW* gene (non-discriminant). 0.5 – 1 µl of the nested PCR products were used for cycle sequencing with 1 µl of SupreDye v1.1 Cycle Sequencing chemistry (BioCat, Heidelberg, Germany) for 25 cycles at 55°C. Isopropanol-precipitated sequencing products were separated on an ABI 3130 capillary sequencer (ThermoFischer/Applied Biosystems, Darmstadt, Germany).

ONT Cas9-based targeted long-read sequencing

High molecular weight (HMW) DNA was isolated from fresh blood using the NEB HMW DNA Extraction Kit (New England Biolabs) and its quality tested by pulsed-field electrophoresis on a CHEFII instrument (Bio-Rad, Munich, Germany). For targeted sequencing of the *OPN1LW-OPN1MW* locus we applied the Oxford Nanopore Cas9-targeted sequencing (nCATS) strategy (Gilpatrick et al. 2020) using the SQK-CS9109 Cas9 Sequencing Kit (Oxford Nanopore Technologies, Oxford, United Kingdom). In brief, 5 µg of HMW DNA (w/o or with prior *Not*I digestion to reduce viscosity) were dephosphorylated for 10 min at 37°C followed by inactivation of the phosphatase for 2 min at 80°C. Dephosphorylated DNA was divided into two portions with each portion subjected to cleavage with 0.125 µM Cas9 ribonucleotide complexes prepared from recombinant Cas9 (Alt-R-Cas9, Integrated DNA Technologies [IDT], Leuven Belgium), tracRNA (IDT) and either pool 1 (BL-715 5'-AAGGACCUACAGCUCAUGGGGUUUUAGAGCUAUGCU-3' and CHOP_267 5'-CUGAAAUGCCCCAUGUCCGGGUUUUAGAGCUAUGCU-3') or pool 2 (IDT_855 5'-CGCAAGCAAGUCAAGGGAUCGUUUUAGAGCUAUGCU-3' and CSPO_586 5'-UCUGGACACUCUGUCGACGGGUUUUAGAGCUAUGCU-3') of crRNAs. *In vitro* cleavage was done for 60 min at 37°C, followed by 3' A-addition with Taq Polymerase for 5 min at 72°C and subsequent adaptor ligation for 10 min at room temperature. Samples were purified using AmpPure beads (0.3x) and washed once with LF buffer and once with SF buffer (Oxford Nanopore Technologies). The purified library was eluted in 18 µl elution buffer for 16 h at room temperature.
Two libraries were prepared and the complete eluted Cas9-enriched library was loaded each on a single MinION flow cell FLO-MIN106D R9.4.1 and run for 72 hours. Basecalling of the raw data used Guppy v.6.3.9 with the dna_r9.4.1_promethion_384 (HAC) model. In a primary alignment step, all reads were mapped to the complete GRCh38 reference genome using minimap2 (Li 2018). We then used the four specific sequences for the Cas9-based excision to define a target region, with coordinates chrX:154,143,640-154,295,680 (hg38). The alignment of reads mapping inside this target region was refined using a prefix alignment approach with edlib (Šošic & Šikic 2017) called from a custom python script.

Supplementary References

Gilpatrick, T., Lee, I., Graham, J.E., Raimondeau, E., Bowen, R., Heron, A., Downs, B., Sukumar, S., Sedlazeck, F.J. & Timp, W. Targeted nanopore sequencing with Cas9-guided adapter ligation. *Nat. Biotechnol.* 38, 433-438 (2020.)

Li, H. Minimap2: pairwise alignment for nucleotide sequences. *Bioinformatics* **34**, 3094-3100 (2018).

Šošic, M. & Šikic, M. Edlib: a C/C ++ library for fast, exact sequence alignment using edit distance. *Bioinformatics* **33**, 1394-1395 (2017).

Wolf, S., Sharpe, L.T., Schmidt, H.J., Knau, H., Weitz, S., Kioschis, P., Poustka, A., Zrenner, E., Lichter, P. & Wissinger, B. Direct visual resolution of gene copy number in the human photopigment gene array. *Invest. Ophthalmol. Vis. Sci.* **40**, 1585-1589 (1999).

```
154,239,648      GGATCACGAGGTCAGGAGATCGAGACCATCCTGGCCAACACGGTGAAACC
                 ||||||||||||||||||||||||||||||||||||||||||||||||||
154,165,373      GGATCACGAGGTCAGGAGATCGAGACCATCCTGGCCAACACGGTGAAACC

154,239,698      CCGTCTCTACTAAAAATACAAAAATTAGCCGGGCGTGGTGGTGCGCGCCT
                 |||||||||||||||
154,165,423      CCGTCTCTACTAAAA-----------------------------------

154,239,748      GTAGTCCCAGCTACTCGGGAGGCTGAGGCAGGAGAATGGCGTGAACCCGG
154,165,437      --------------------------------------------------

154,239,798      GAGGCGGAGCTTGCAGTGAGCCGAGATCACGCCACTGCACTCCAGCCTGG
154,165,437      --------------------------------------------------

154,239,848      GTGACAGAACGAGACTCCGTCTCAAAAAAAAAAAAAAAAATTGTTCCAAAA
154,165,437      --------------------------------------------------

154,239,898      CATTTTCATCACTGCCTCGAAAAAATACTATTTAGCAGTCACTCCCCATT
154,165,437      --------------------------------------------------

154,239,948      CCCCATCACCCCAGTCCCTGGTAACCACCAATCTGCTTTCTGACTTGAGT
154,165,437      --------------------------------------------------

154,239,998      AGCTTCTTATCTCTGAACCTCTACCAGCAATTTATGAATGAGAGATGGAC
154,165,437      --------------------------------------------------

154,240,048      AAGACCATCAGGTATGTGGAGACGGTCCTAGAGAAGCTGCTAGAAAGTGA
154,165,437      --------------------------------------------------

154,240,098      CAGACAACCAGAGTAAGGGGTAGTGGAGCACTGTAATGCTGTGGAGAGGA
154,165,437      --------------------------------------------------

154,240,148      GAGGGCAGCAGGAATGACAAACCCAGGAGGCATTGACTCAGGGAAAGAAG
154,165,437      --------------------------------------------------

154,240,198      AGGAAGGCAGGGTCAGGTCGAGATCTTGGCCAAATCCCTGAGCTCATCAA
154,165,437      --------------------------------------------------

154,240,248      CCTAGGGTGCTCCATTGATGAATTTATCAAGAGTCATTGGCCGGGAGTGG
154,165,437      --------------------------------------------------

154,240,298      TGGCTCACGCCTGTAATCACAGCACTTTGGGAGGCCAAGGCAGGTGGATC
154,165,437      --------------------------------------------------

154,240,348      ATTTGAGATTAGGAGTTGGAGACCAGCCTGGCCAACATGATGAAACCCCG
154,165,437      --------------------------------------------------

154,240,398      TCTCTACTAAAATATGCAAAAATTAGCTGGGCGTGGTGGTGCATGCCTGT
                             ||||||||||||||||||||||||||||||||||||||
154,165,437      ------------TATGCAAAAATTAGCTGGGCGTGGTGGTGCATGCCTGT

154,240,448      AATCTCAGCT
                 ||||||||||
154,165,476      AATCTCAGCT
```

Supplementary Figure 1: Sequence of the 697 bp SDIns polymorphism (highlighted in yellow) and alignment of the flanking sequence with an SD copy (SD1) sequence lacking the insertion. Coordinates based on the GRCH/hg38 assembly.

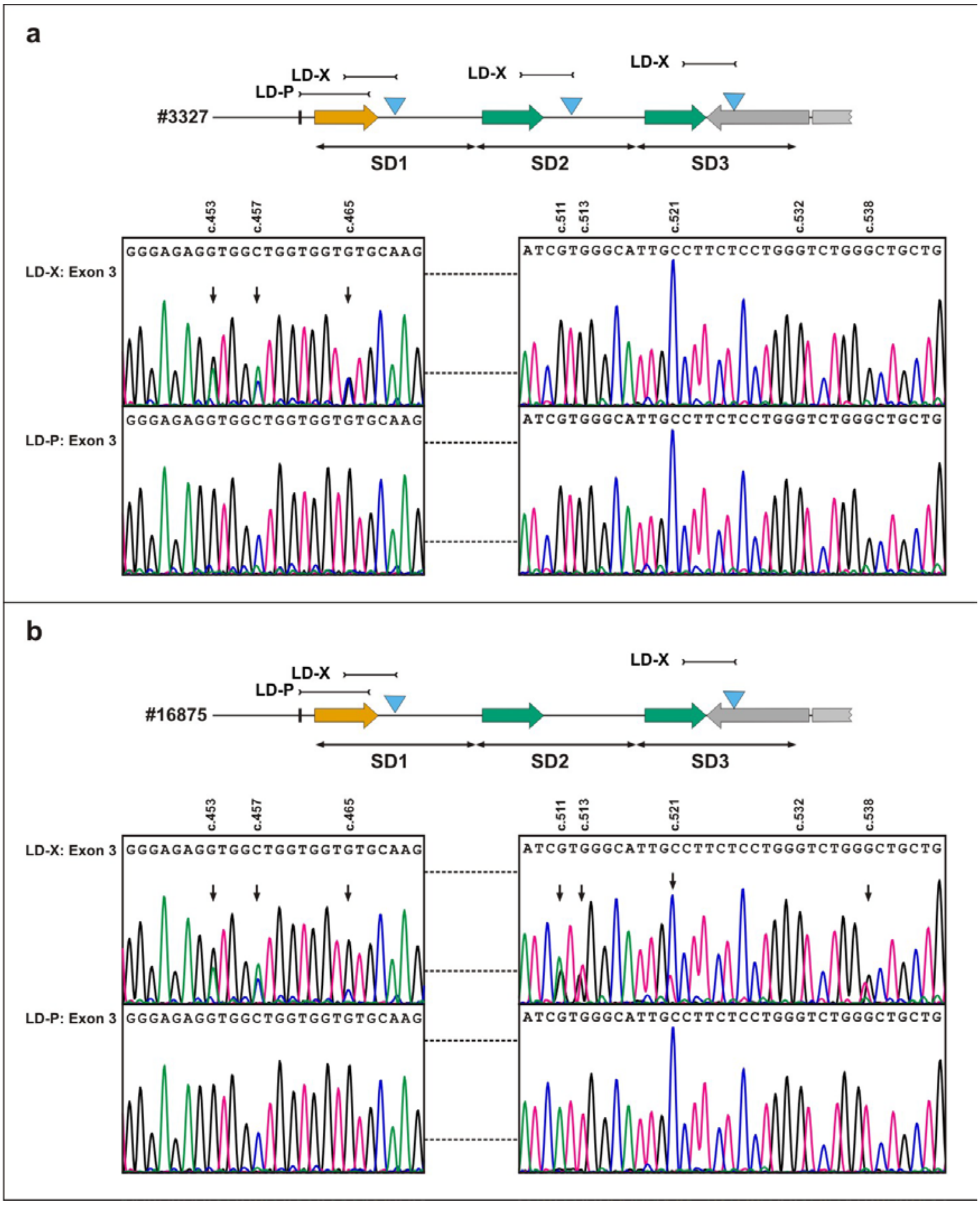

Supplementary Figure 2: Ambiguous exon 3 sequences based on the design of LD PCR amplicons. Exon 3 sequences for proband #3327 (A) and proband #16875 (B), both with a clinical diagnosis of cone dysfunction potentially linked to exon 3 haplotype associated splicing defects. Exon 3 sequences were obtained for the LD PCR amplicon LD-X (as proposed by Haer-Wigman and co-workers) in comparison with those obtained for LD PCR amplicons LD-P (specific for the most proximal gene copy). Top: Deduced structure of the *OPN1LW-OPN1MW* gene array. SD1, SD2, and SD3 indicate the number and extent of individual segmental duplications forming the opsin gene cluster. The blue triangles indicate the presence and localization of the 697 bp SDIns insertion. Bottom: Sanger sequence traces for two segments of exon 3 as obtained from the different LD PCR amplicons. The eight common variant sites in exon 3 which jointly form haplotypes, some of which induce splicing defects, are indicated above the chromatograms. Arrows indicate overlaid sites in the sequence obtained from the LD-X amplicon.

Supplementary Table 1: Oligonucleotide Primer Sequences

Name	Orientation	Sequence (5'-3')	Amplicon / Application
LCR1F	Forward	CACCCTTCTGCAAGAGTGTGGG	LD-PCR (LD-P)
RGCP2-5R	Reverse	*GCGGACTAGT*GATCTGCTGATGGTGTTGCTTA	LD-PCR (LD-P)
FEO 27	Forward	ACAAACCCCACCCGAGTTAG	LD PCR (LD-D)
RGCP_FODA	Forward	TGCAACAAAGCCCTAAGCAGAT	LD PCR (LD-Ds, LD-SDIns)
RGCP_ODA-R	Reverse	GCTCGCCCTGTGTCTGAAATG	LD PCR (LD-D, LD-Ds)
OPN1-IG2R	Reverse	GTGACTGCCCAAAGACATGTTG	LD PCR (LD-LW, LD-SDIns)
RCP-4F	Forward	CATCCCACTCGCTATCATCA	LD PCR (LD-LW)
79R-F	Forward	GATCTTTGCGTACTGCGTCTGCTGG	LD PCR (LD-LW)
HW099-820	Forward	AGGTGTAGAGCCCTAGCAAAC	LD PCR (LD-X), Haer-Wigman et al. 2022
HW099-821X	Reverse	*TC*TCTCATTCATAAATTGCTGGTA	LD PCR (LD-X), Haer-Wigman et al. 2022
FEO 201	Forward	TCCAACCCCCGACTCACTATC	Exon 5 nested PCR
FEO 202	Reverse	ACGGTATTTTGATGTGGATCTGCT	Exon 5 nested PCR
Ex 4F/FEO 27	Forward	ACAAACCCCACCCGAGTTAG	Exon 4 nested PCR & Sequencing
Ex 4R/FEO 26	Reverse	AGGAGTCTCAGTGGACTCAT	Exon 4 nested PCR & Sequencing
Ex 3F	Forward	TGGTGGAAAGAAAGATGTCG	Exon 3 nested PCR & Sequencing
Ex 3R	Reverse	GCCCAGAGAAAGGAAGTGATT	Exon 3 nested PCR & Sequencing
Ex 5 Seq-F	Forward	AATCCACCCAGAAGGCAGA	Exon 5 Sequencing
Ex 5 Seq-R	Reverse	GGGTTGTAGATAGTGGCAC	Exon 5 Sequencing
OPN1-23_QF	Forward	AGACAACCCCAGAACAGTAGG	qPCR (123 bp), OPN1LW & OPN1MW copy number
OPN1-23_QR	Reverse	AGGATCTGGGAATTGTGTCTCC	qPCR (123 bp), OPN1LW & OPN1MW copy number
OPN1-45_QF	Forward	GTGATTCCTTTGGGACTGTACC	qPCR (118 bp), OPN1LW & OPN1MW copy number
OPN1-45_QF	Reverse	TGTTCCCTTAGTGGACTCCTC	qPCR (118 bp), OPN1LW & OPN1MW copy number
OPN1ins-QF	Forward	GCTGCTAGAAAGTGACAGACAAC	qPCR (97 bp), SDIns copy number
OPN1ins-QR	Reverse	TGCCTCCTGGGTTTGTCATTC	qPCR (97 bp), SDIns copy number
SDC4_F	Forward	CAGGGTCTGGGAGCCAAGT	qPCR, autosomal copy number control
SDC4_R	Reverse	GCACAGTGCTGGACATTGACA	qPCR, autosomal copy number control

Supplementary Table 2: List of reagents and Suppliers

**Reagent / Kit**	**Supplier**	**Catalog-No.**
Quantitect SYBR Green PCR Kit	Qiagen	204143
X80-B1 Probe Mix	MRC-Holland	Pre-marketing
SALSA MLPA EK1 Reagent Kit	MRC Holland	EK1-FAM-I
MLPA Control Sample	MRC Holland	SD083-S01
NEB LongAmp HotStart Taq 2 x Master Mix	New England Biolab	M0533L
Roche Expand Long Range dNTPack	Merck	4829034001
Restriction Endonuclease, *Bam*HI	New England Biolab	R0136
Restriction Endonuclease, *Not*I	New England Biolab	R0189
Restriction Endonuclease, *Sal*I	New England Biolab	R0138
λ-DNA/*Hind*III cleaved (size standard)	ThermoFisher/Fermentas	SM0103
λ-DNA/*Hind*III+EcoRI cleaved (size standard)	ThermoFisher/Fermentas	SM0191
GeneRuler 1 kb DNA Ladder (size standard)	ThermoFisher/Fermentas	SM0311
Qiagen Multiplex PCR Kit (100)	Qiagen	206143
Taq Polymerase	GenAxxon	M3001.X
SupreDye v1.1 Cycle Sequencing Kit	BioCat	060008-ADS
Illustra ExoPro Star 1-Step	Cytiva	US77720
NEB HMW DNA Extraction Kit	New England Biolabs	T3060S/L
Alt-R-S.p. Cas9 nuclease V3	Integrated DNA Technologies	1081058
tracRNA	Integrated DNA Technologies	1072533
crRNA synthesis	Integrated DNA Technologies	custom
Cas9 Sequencing Kit	Oxford Nanopore Technologies	SQK-CS9109
AMPure beads	Beckman-Coulter	B23317
MinIon Flow Cell	Oxford Nanopore Technologies	FLO-MIN106D R9.4.1
Oligonucleotides	Merck/Genosys, Integrated DNA Technologies, Eurofins	custom
